# PERIOPERATIVE CHEMOTHERAPY, ADJUVANT CHEMOTHERAPY AND ADJUVANT CHEMORADIOTHERAPY IN THE SURGICAL TREATMENT OF GASTRIC CANCER IN A HOSPITAL OF THE BRAZILIAN UNIFIED HEALTH SYSTEM

**DOI:** 10.1590/0102-6720202400017e1810

**Published:** 2024-07-01

**Authors:** Eduardo Marcucci PRACUCHO, Renato Morato ZANATTO, Júnea Caris de OLIVEIRA, Luiz Roberto LOPES

**Affiliations:** 1 Hospital Amaral Carvalho, Department of Abdominal and Pelvic Surgery – Jaú (SP), Brazil;; 2 Universidade Estadual de Campinas, Faculty of Medical Sciences, Department of Surgery – Campinas (SP), Brazil.

**Keywords:** Gastric Cancer, Adjuvant Chemotherapy, Neoadjuvant Therapy, Chemoradiotherapy, Survival Analysis, Prognostic Factor, Câncer gástrico, Quimioterapia Adjuvante, Terapia Neoadjuvante, Quimiorradioterapia, Análise de Sobrevida, Fator Prognóstico

## Abstract

**BACKGROUND:**

Despite the preference for multimodal treatment for gastric cancer, abandonment of chemotherapy treatment as well as the need for upfront surgery in obstructed patients brings negative impacts on the treatment. The difficulty of accessing treatment in specialized centers in the Brazilian Unified National Health System (SUS) scenario is an aggravating factor.

**AIMS:**

To identify advantages, prognostic factors, complications, and neoadjuvant and adjuvant therapies survival in gastric cancer treatment in SUS setting.

**METHODS:**

The retrospective study included 81 patients with gastric adenocarcinoma who underwent treatment according to INT0116 trial (adjuvant chemoradiotherapy), CLASSIC trial (adjuvant chemotherapy), FLOT4-AIO trial (perioperative chemotherapy), and surgery with curative intention (R0 resection and D2 lymphadenectomy) in a single cancer center between 2015 and 2020. Individuals with other histological types, gastric stump, esophageal cancer, other treatment protocols, and stage Ia or IV were excluded.

**RESULTS:**

Patients were grouped into FLOT4-AIO (26 patients), CLASSIC (25 patients), and INT0116 (30 patients). The average age was 61 years old. More than 60% of patients had pathological stage III. The treatment completion rate was 56%. The pathological complete response rate of the FLOT4-AIO group was 7.7%. Among the prognostic factors that impacted overall survival and disease-free survival were alcoholism, early postoperative complications, and anatomopathological status pN2 and pN3. The 3-year overall survival rate was 64.9%, with the CLASSIC subgroup having the best survival (79.8%).

**CONCLUSIONS:**

The treatment strategy for gastric cancer varies according to the need for initial surgery. The CLASSIC subgroup had better overall survival and disease-free survival. The INT0116 regimen also protected against mortality, but not with statistical significance. Although FLOT4-AIO is the preferred treatment, the difficulty in carrying out neoadjuvant treatment in SUS scenario had a negative impact on the results due to the criticality of food intake and worse treatment tolerance.

## RESUMO

**RACIONAL:**

Apesar da preferência pelo tratamento multimodal para o câncer gástrico, o abandono do tratamento quimioterápico bem como a necessidade de cirurgia “upfront” em pacientes obstruídos traz impactos negativos para o tratamento. A dificuldade de acesso ao tratamento em centros especializados no Sistema Único de Saúde (SUS) é um agravante.

**OBJETIVOS:**

Identificar vantagens, fatores prognósticos, complicações e sobrevida de terapias neoadjuvantes e adjuvantes no tratamento do câncer gástrico no cenário do SUS.

**MÉTODOS:**

Estudo retrospectivo incluindo 81 pacientes com adenocarcinoma gástrico submetidos a tratamento segundo os protocolos INT0116 (quimiorradioterapia adjuvante), CLASSIC (quimioterapia adjuvante), FLOT4-AIO (quimioterapia perioperatória) e cirurgia com intuito curativo (ressecção R0 e linfadenectomia D2) em um único centro oncológico entre 2015 e 2020. Indivíduos com outros tipos histológicos, coto gástrico, câncer de esôfago, outros protocolos de tratamento e estádio Ia ou IV foram excluídos.

**RESULTADOS:**

Os pacientes foram distribuídos em: FLOT4-AIO (26 pacientes), CLASSIC (25 pacientes) e INT0116 (30 pacientes). A média de idade foi 61 anos. Mais de 60% dos pacientes apresentaram estádio III patológico. A taxa de completude do tratamento foi 56%. A taxa de resposta patológica completa do grupo FLOT4-AIO foi 7,7%. Dentre os fatores prognósticos que impactaram a sobrevida global e sobrevida livre de doença tivemos etilismo, complicações pós-operatórias precoces, status anatomopatológico pN2 e pN3. A taxa de sobrevida global em 3 anos foi 64,9% sendo o subgrupo CLASSIC com melhor sobrevida (79,8%).

**CONCLUSÕES:**

A estratégia de tratamento do câncer gástrico varia de acordo com a necessidade de cirurgia inicial. O subgrupo CLASSIC apresentou melhor sobrevida global e sobrevida livre de doença. O esquema INT0116 também protegeu contra a mortalidade, mas não com significância estatística. Apesar do FLOT4-AIO ser o tratamento de escolha, a dificuldade na realização da neoadjuvância no âmbito do SUS impactou negativamente nos resultados devido à criticidade da ingesta alimentar e à pior tolerância ao tratamento.

## INTRODUCTION

Gastric cancer (GC) is a malignant neoplasm of great relevance both around the world and in Brazil. It is the fifth most common cancer, the sixth most prevalent, and the fourth cause of death from cancer worldwide. In Brazil, it is the fourth most common cause among men and the sixth among women^20^.

The results of treating this tumor in its early stages are encouraging. However, since the majority of patients present with advanced disease at the time of diagnosis, although surgery improves the quality of treatment, with adequate morbidity and mortality rates, half of the patients still experience tumor recurrence, creating a demand for research into multimodal treatment for gastric adenocarcinoma^11^.

In 2001, Intergroup 0116 (INT-0116) published the first study that demonstrated the benefit of multimodal treatment combining adjuvant chemotherapy and radiotherapy, showing an increase in the overall survival of treated patients compared to the group treated with surgery alone^16^. However, critical to this work was that 80% of patients did not undergo adequate lymphadenectomy. Still, years later, the same group published their results from ten years of follow-up and showed benefits in overall survival^26^. After the encouraging results from the United Kingdom with the Medical Research Council Adjuvant Gastric Infusional Chemotherapy (MAGIC Trial)^8^ through neoadjuvant chemotherapy, the CLASSIC trial^2^ from South Korea also showed benefit from adjuvant chemotherapy, until FLOT4-AIO^1^ displaced the MAGIC trial, being the scheme of choice in most of the West.

Despite achieving good tolerance in neoadjuvant treatment, the FLOT4-AIO regimen presents high rates of abandonment, toxicity, and hospitalizations secondary to adjuvant chemotherapy^10^. Furthermore, the need for upfront surgery in obstructed patients impacts treatment outcomes. This fact is aggravated in the scenario of the Brazilian Unified National Health System (SUS) since we find delays in diagnosis, difficulty in accessing treatment in tertiary centers, and lack of transportation to attend appointments scheduled during specialized treatment^5,25^.

The criticism for most of the published works is due to the heterogeneity of the samples, mixing gastric cancer with cancer of the esophagus and esophagogastric junction, as well as the lack of studies that compare neoadjuvant and adjuvant regimens^6^.

Therefore, this study sought to identify the advantages and disadvantages of using neoadjuvant and adjuvant therapies in the treatment of gastric cancer in the SUS scenario.

## METHODS

This is a retrospective cohort study from a single cancer center within the SUS setting. There were 81 patients suffering from gastric cancer who underwent R0 surgical resection, D2 lymphadenectomy^13^, and multimodal treatment schemes: INT0116 (30 patients), CLASSIC (26 patients) and FLOT4-AIO (25 patients), between 2015 and 2020. Cases of esophageal cancer, esophagogastric junction (EGJ) Siwert I and II cancer^24^, gastric stump cancer, other multimodal treatment regimens, and T1a or M1 were excluded.

The study was developed with its own financing and approved by the Ethics and Research Committee of Hospital Amaral Carvalho with a Certificate of Presentation for Ethical Appreciation (CAAE) under number 62132816.7.0000.5434.

The variables analyzed were age, symptoms, comorbidities, multimodal treatment regimens (FLOT4-AIO, INT0116, and CLASSIC), toxicity, treatment completeness, postoperative morbidity according to the Clavien-Dindo classification^9^, anatomopathological analysis, complete pathological response rate, and overall and disease-free survival.

Statistical analysis was carried out by measuring quantitative variables expressed by measuring the mean with the assessment of dispersion through the standard deviation and the median through the interquartile range. To compare groups with numerical variables and normal distribution, the Analysis of Variance (ANOVA) test was used, while for those without normal distribution, we opted for the Kruskal-Wallis test. In the case of categorical variables, we employed Pearson’s chi-square (χ²) test to compare groups of proportional sizes and Fisher’s exact test for non-proportional groups. To evaluate the association of each variable with overall and disease-free survival, we used univariate Cox regression analysis. The analysis of overall survival and disease-free survival was performed by applying the Kaplan-Meier method and comparison of curves, using the log-rank test. The variables that had p<0.050 by the log-rank test were selected for multivariate Cox regression analysis in order to ascertain the real impact of each variable on overall and disease-free survival.

## RESULTS

The average age was 58.5 years for the FLOT4-AIO group, 65.4 years for the INT0116 group, and 59.2 years for the CLASSIC group, with disproportion between the groups in the ANOVA evaluation (p-value [p]=0.014, p<0.050). Regarding the distribution between sexes, the INT0116 (73.3%) and CLASSIC (76.0%) groups had a greater number of male patients, unlike the FLOT4-AIO group (46.2%) which showed a predominance of women. This distribution also showed statistical significance (p=0.042, p<0.050). The majority of patients (55.5%) had pathological stage III, with weight loss (84.7%), and impaired food intake (86.4%) at the first consultation. On average, 64.2% of patients experienced gastrointestinal tract toxicity, and 44.0% were unable to complete multimodal treatment. In the subgroup analysis, we found adherence of 80% for neoadjuvant FLOT4-AIO and 41% adjuvant, 83% for INT0116, and 52% for the CLASSIC group. The pathological complete response rate of the FLOT4-AIO group was 7.7%, but the best survival of this subgroup did not show statistical significance.

The type of multimodal treatment used was also associated with the impact on overall survival and disease-free survival, with the CLASSIC regimen showing the best outcome (hazard ratio [HR] 0.26; 95%CI 0.08–0.81; p=0.019, p<0.050. The INT0116 regimen also protected against mortality, but the p-value was not significant (HR 0.69; 95%CI 0.28–1.71; p=0.430, p>0.050).

Regarding postoperative morbidity, the average number of patients who presented early surgical complications was 40.7%, of which 96.3% were mild, that is, Clavien-Dindo type 1 and 2^9^. However, in the multivariate Cox regression analysis, early postoperative complications caused poor overall survival (HR 2.47; 95%CI 1.21–5.04; p=0.012, p<0,050).

The average overall survival of all 81 patients was 44.9 months, and the average disease-free survival was 37.8 months, with the peritoneum being the most frequent site of recurrence (Table 1).

**Table 1:** Distribution of variables related to relapse and disease-free survival according to the treatment scheme.

Factors	N	OS (CI) 36 months	p-value	DFS (CI) 36 months	p-value
Global	81	64.9% (53–74)		61.9% (50–72)	
FLOT4-AIO	26	57.7% (37–74)	0.058	51.1% (29–69)	0.050
CLASSIC	30	58.6% (39–74)		55.3% (36–71)	
INT0116	25	79.8% (58–91)		79.6% (58–91)	

OS: overall survival; CI: confidence interval; DFS: disease-free survival.

The median used to calculate overall and disease-free survival in this study was 36 months, since the median of the FLOT4-AIO group was 37 months, enabling the comparison of the three groups in a balanced way regarding their outcomes. Therefore, the overall survival median of the study was 64.9%, and the results of each subgroup are shown in Table 2.

**Table 2:** Overall survival and median disease-free survival of subgroups at 36 months.

Variable	FLOT4-AIO	INT0116	CLASSIC	p-value
	26 (%)	30 (%)	25 (%)	
Relapse				
No	15 (57.7)	21 (70.0)	21 (84.0)	0.120
Yes	11 (42.3)	9 (30.0)	4 (16.0)	
Relapse site				
Lymph node	2 (18.1)	1 (11.1)	2 (50.0)	0.101
Liver	2 (18.1)	2 (22.2)	2 (50.0)	
Peritoneum	6 (54.8)	7 (66.7)	0 (0)	
Other	1 (9)	0 (0)	0 (0)	
Disease-free survival				
Median (months)	29.7	46.6	43.4	0.013
Death				
No	13 (50)	15 (50)	20 (80)	0.040
Yes	13 (50)	15 (50)	5 (20)	

**Figure 1:**
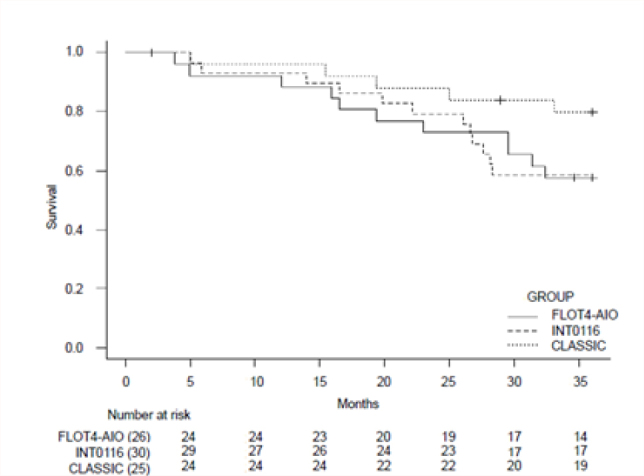
Median overall survival of subgroups at 36 months. com diferença estatisticamente significativa (p = 0,04).

**Figure 2:**
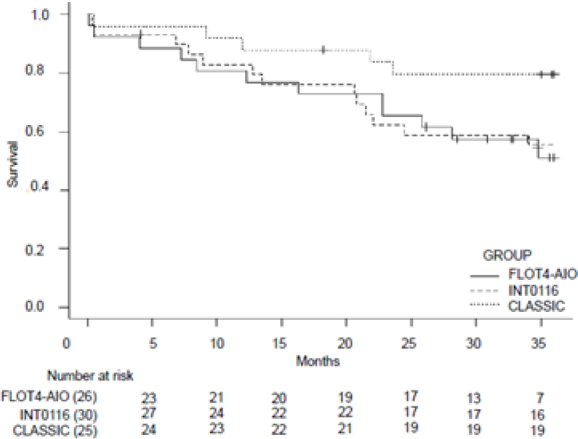
Median disease-free survival of subgroups at 36 months.

The CLASSIC subgroup presented overall survival and disease-free survival curves that were superior to the other subgroups (Figures 1 and 2).

The distribution of the incidence of alcoholism, early surgical complications, and the anatomopathological status pN2 and pN3 can be seen in Table 3. These were the factors that showed a statistically significant difference in the overall survival of patients (Table 4).

**Table 3:** Distribution by subgroups of factors that impacted overall survival.

Variable	FLOT4-AIO	INT0116	CLASSIC	p-value
	26 (%)	30 (%)	25 (%)	
Alcoholism				
No	23 (88.5)	27 (90.0)	24 (96.0)	0.695
Yes	3 (11.5)	3 (10.0)	1 (4.0)	
Early surgical complication				
No	13 (50.0)	19 (63.3)	16 (64.0)	0.506
Yes	13 (50.0)	11 (36.7)	9 (36.0)	
pN2	8 (30.8)	7 (23.3)	6 (24.0)	0.981
pN3	2 (7.7)	7 (23.3)	5 (20.0)	0.981

pN2, pN3: lymph node staging.

**Table 4:** Factors that impacted overall survival.

Factors	Hazard ratio	95%CI	p-value
Alcoholism	3.56	1.24–10.25	0.0186
Early surgical complication	2.48	1.22–5.04	0.0123
pN3a	6.20	2.14–18.00	0.0007
pN3b	15.32	2.85–82.23	0.0014

pN3a, pN3b: lymph node staging; CI: confidence interval.

## DISCUSSION

Adherence to multimodal treatment and its completeness has an impact on overall survival. The completion rate of the INT0116, CLASSIC, and FLOT4-AIO studies were 65.0, 66.0, and 40.5%, respectively, compared to 56.0% in our study^14^. It is worth noting that the treatment completion rate in our study, both in the CLASSIC and INT0116 subgroups, was higher than that of the FLOT4-AIO subgroup, as observed in the literature. Both postoperative surgical morbidity and the toxicity of adjuvant chemotherapy contributed to the high dropout rates in the FLOT4-AIO group, negatively affecting the outcome of this group.

Complete pathological response is also an important prognostic factor in the treatment of GC^7^. However, there is a bias in this analysis among examiners depending on the type of classification used. The Mandard classification assesses the degree of post-neoadjuvant fibrosis^18^ while the Becker classification assesses the percentage of tumor cells remaining post-neoadjuvant^3,17^. In our sample, the modified Ryan scale was used, which has been recommended by the College of American Pathologists as it more objectively assesses the viability of post-neoadjuvant tumor cells^19,21,22^. The pathological complete response rate was 7.7% compared to 16% in the FLOT4-AIO study. Both overall and disease-free survival in this subgroup had better results (100% and 50%, respectively), although without statistical significance. We attribute the observed differences to the discrepancy between our sample (25 patients undergoing FLOT4-AIO) and the FLOT4-AIO study sample (356 patients).

Although only half of the patients were able to complete the adjuvant CLASSIC regimen in this study, it was still the one that demonstrated better survival, suggesting that surgery with margins and adequate lymphadenectomy has a great influence on increasing overall and disease-free survival^23^.

Currently, FLOT4-AIO is the treatment of choice in the European Society for Medical Oncology (ESMO), National Comprehensive Cancer Network (NCCN), and Brazilian Group of Gastrointestinal Tumors (GTG)^15^. The CLASSIC subgroup, in our study, obtained better overall and disease-free survival results with statistical power than FLOT4-AIO. We attribute this superiority to the higher treatment abandonment rate in the FLOT4-AIO group compared to the CLASSIC group, since both underwent the same level of surgical quality with no statistical difference in surgical morbidity.

The INT0116 study is still used today, mainly for patients who underwent inadequate surgery with an amount of less than 15 lymph nodes in the surgical specimen^4^. In our study, all patients underwent D2 lymphadenectomy and the INT0116 subgroup was also superior to FLOT4-AIO, but without statistical power.

Perioperative treatment, despite being currently the treatment of choice due to the FLOT4-AIO results, presents difficulties regarding the completion of cycles, especially adjuvants due to toxicity, presenting treatment abandonment rates higher than CLASSIC and INT0116 treatment. Furthermore, due to the delay in referring these patients to reference centers, the option for upfront surgery due to precarious food intake upon admission favors the use of adjuvant therapies^12^.

## CONCLUSION

Patients undergoing adjuvant chemotherapy treatment had better overall survival and disease-free survival, which can be a valuable tool in cases of upfront surgery.

Therefore, there is still room for adjuvant therapies, especially in the SUS scenario where upfront surgery is often necessary. However, more studies with larger samples are needed comparing neoadjuvant and adjuvant regimens in order to achieve a better analysis of the advantages and disadvantages of these two strategies.
